# Perceptions of public health education among medical students in Ireland: A qualitative study

**DOI:** 10.1016/j.puhip.2026.100730

**Published:** 2026-01-14

**Authors:** A. Malakhveitchouk, P. White, P. Barrett

**Affiliations:** a School of Medicine, University College Cork, Cork, Ireland; b University of Toronto, 27 King's College Cir, Toronto, Canada, ON, M5S 1A1; c HSE Public Health, Dr Steevens’s Hospital, Dublin 8, Ireland; d Department of Public Health HSE South West, St. Finbarr's Hospital, Cork, Ireland; e Irish Centre for Maternal and Child Health Research, University College Cork, Cork, Ireland; f School of Public Health, University College Cork, Cork, Ireland

**Keywords:** Medical student perceptions, Education, Recruitment

## Abstract

**Objectives:**

This study seeks to explore the perceptions, understanding and expectations held by medical students studying in Ireland towards public health (PH) education and the practice of public health medicine (PHM).

**Study design:**

Qualitative semi-structured interviews.

**Methods:**

Participants were recruited from all medical universities in Ireland using purposive and snowball sampling. Qualitative semi-structured interviews were conducted and transcribed. Thematic analysis was performed using the Braun and Clarke method to identify semantic and latent themes. Transcripts were read repeatedly for familiarization with the data, following which initial codes were created. Related codes were grouped into categories and these were subsequently reviewed to generate themes.

**Results:**

13 medical students in their penultimate and final year across five Irish universities were recruited. Three key themes were generated from the analysis. 1) Variations in perceived relevance of PH to medical training. Students articulated mixed views on the importance of PH in the medical curriculum, as well as misconceptions around the role of PH physicians. 2) Deprioritization of PH education during clinical training. Participants perceived that PH was deprioritized by institutions and by students themselves. 3) Limited exposure to PH in practice. The reduced visibility of PH practitioners, and limited exposure to PH workplaces during clinical training deters students from considering PHM as a future career.

**Conclusions:**

There is a need to address students’ lack of understanding and misconceptions relating to PH practice within the medical curriculum in Ireland and to provide more practical opportunities for exposure to PHM.

## 1 Introduction

Public Health Medicine (PHM) has often been perceived to be a lesser-known medical specialty, sometimes misunderstood by both the medical profession and the wider public. In Ireland, PHM physicians have only recently been awarded Consultant status for the first time in 2021, putting them on an equal standing with their medical peers [1]. This change occurred as part of wide-ranging public health reforms arising from the Covid-19 pandemic, which publicly exposed global healthcare vulnerabilities, and underscored the essential role of public health (PH) physicians and other PH practitioners in crisis response. However, despite improvements in the overall public understanding of the role of PHM physicians, further efforts are needed to promote this specialty and to address misconceptions of the role. For several years, leading medical organizations have called for stronger connections between medical curricula in university settings and PH practitioners [[2], [3], [4], [5]]. This requires improved understanding of the role of PH practitioners, including PH physicians.

Medical education traditionally emphasizes pathophysiology, disease diagnosis, and individual patient care, while PH education often focuses on broader health determinants, disease prevention, and population health. Although relatively few medical graduates may choose to ever specialize in PHM, all physicians may benefit from achieving core PH knowledge and competencies to enable them to advocate for their own patients, to promote health equity, and to prevent disease. Calls for more PH education in medical schools date back 150 years, and integrating PH in to modern medical curricula is likely to boost visibility of the specialty while aiding future recruitment of public health physicians [6].

In Canada, PH integration in to medical education has improved since the early 2000s, spurred by crises like the SARS and Walkerton outbreaks, leading to greater inclusion of PHM in exams and professional frameworks [7]. The U.S. and U.K. have also reformed their approach to medical training by enhancing the prominence of PH education, and this is reflected in updates to the Graduate Medical Education curriculum, and reports from the General Medical Council UK respectively [[7], [8], [9]].

In Ireland, several reports have examined the extent of PH integration into medical curricula. A 2003 report by the Irish Medical Council claimed that Irish medical schools dedicated less than 7 % of instruction time to PH education, and in some cases, this was less than 1 % of instruction time, with virtually no student exposure to the delivery of PHM in community settings [10]. An updated report in 2007 further stressed the need to deliver PH teaching in community-based settings [11]. A 2006 report of the Working Group on Undergraduate Medical Education and Training described barriers to quality medical education in Ireland which included inadequate exposure to community-based specialties including PHM [12]. While these reports provide some insight and context, they are relatively outdated. Few recent studies exist on medical students' perceptions of PH education, and to our knowledge, none have been conducted in Ireland to date.

## 2 Methods

Penultimate and final year medical students in Ireland were invited to participate in semi-structured qualitative interviews between December 2022 and April 2023. A topic guide was developed based on a scoping literature review on the integration of PHM into medical curricula. The scoping review identified a number of recurring themes internationally: limited visibility of PHM in medical curricula, student misconceptions about PHM roles, and the lower prioritization of PHM relative to biomedical sciences.

Interviews were conducted via Zoom or MS Teams and recorded for transcription.

Participants were recruited using purposive and snowball sampling to seek diversity in age, gender, university attended, programme type (i.e. direct entry undergraduate medicine, and graduate-entry postgraduate medicine), and baseline level of interest in PH (assessed by asking if participants had completed electives in PHM). Programme directors at all Irish medical universities were contacted to promote the study among their own students. Participants were also recruited through medical student social media groups, but avoiding those where the membership was based on a pre-specified interest in PH (i.e. Public Health Societies). Students who expressed interest in participating received an information leaflet and consent form before scheduling an interview with the lead researcher (AM).

We aimed to recruit a sample of 12–15 participants. We were guided by the principles of information power, given the wide-ranging, exploratory nature of our which study, which had broad aims [13]. Recruitment ceased when we reached this sample and we considered that we had achieved information power at that point.

Ethical approval was obtained from the Social Research Ethics Committee and the Research and Postgraduate Affairs Committee at University College Cork. Written informed consent from all participants was collected at the start of each interview.

All interviews lasted 20–40 min and were transcribed using Otter AI automated transcription software. Thematic analysis followed Braun and Clarke’s framework [12]. One researcher (AM) conducted manual coding using NVIVO software, with two transcripts audited by a second researcher (PB) to ensure consistency and face validity. Transcripts were read repeatedly for familiarization with the data, following which initial codes were created. A codebook was subsequently created and applied across the interviews. Related codes were grouped into categories and these were subsequently reviewed to generate themes. This was an iterative process, repeated until consensus was achieved with the generated themes.

Themes and subthemes were iteratively refined, and categories were reviewed by a third team member (PW) to minimize bias and enhance trustworthiness of the findings.

## 3 Results

There were 13 study participants. The median age was 27 and ages ranged between 22 and 30 years. The majority of the participants were female (n = 8), of white ethnicity (n = 8), were graduate-entry medical students (n = 11), and most were in their penultimate year of study (n = 9). Six participants were Irish nationals, and seven were non-Irish.

No clear differences in views were observed by age, gender, or university. However, graduate-entry students tended to express slightly greater familiarity with core PH concepts, likely reflecting previous academic or work experience.

Three key themes were generated from the analysis.

### 3.1 Theme 1: Variations in perceived relevance of PH to medical training

#### 3.1.1 Subtheme 1.1: Mixed views on relevance of formal PH education in medical curriculum

Students expressed varying views on the relevance and importance of formal PH education in the medical curriculum. Some students strongly supported the inclusion of PH education in the medical curriculum, recognizing its growing relevance, and the potential to harness PH competencies to drive widespread, or lasting changes at societal level, as well as the fundamental role of PH in health service improvement. One participant described how, “as a PH physician …, you make a bigger mark that will last for generations."

In contrast, some students dismissed PH education as less important or appealing to them, with one stating that "It goes under the radar; people aren't interested." A number of participants referred to PH as a “soft science” or "fluffy stuff" compared to other specialties. These views reflected a perception of PH as being less rigorous and undervalued by peers, or indeed by the broader medical community.

#### 3.1.2 Subtheme 1.2: Greater consensus on relevance of public health principles for future clinical practice

The majority of students acknowledged that PH principles were relevant in various aspects of healthcare delivery, specifically highlighting the roles played by general practitioners (GPs) and infectious disease specialists in disease prevention. As one student summarized, “I think one of the most natural combinations for clinical medicine and PH would be in family medicine”

Several students emphasized the importance of disease prevention as a core competency which is relevant to all physicians, irrespective of their specialty. One student stated, “no one should be thinking only about reactive medicine. We should be thinking about prevention as well.”

The prevailing sentiment among students was that all physicians have a role to play in disease prevention, and that public health principles are relevant to all of their future roles. By contrast, a minority of students expressed opposing views that PH education is entirely irrelevant to their future practice.

#### 3.1.3 Subtheme 1.3: Misconceptions about the role of public health physicians

Most participants struggled to grasp the scope of the PH physician’s role in disease prevention, expressing confusion around why physicians would undertake PH-related work. Misunderstandings of the role were common, with some students conflating PHM physicians with GPs or infectious disease consultants. Moreover, perceptions of PHM physicians as academic researchers, without any applied health service role, were common.

When students attempted to describe the role of a PHM physician, their responses primarily encompassed non-clinical activities, with a particular focus on statistical work.

However, a minority of students described broader roles for PHM physicians in applied disease prevention, health policy work and infectious disease control. While some students recognized the role of PHM physicians in disease surveillance, others mentioned roles in guideline creation, leading health promotion campaigns, and responding to health emergencies, emphasizing the potential impact on the public at large.

### 3.2 Theme 2: Deprioritization of PH education during clinical training

#### 3.2.1 Subtheme 2.1: Lesser institutional emphasis within curriculum and assessment

Students reported that PH was typically underemphasized in the medical curriculum and formal assessments. Several participants described their institution’s PH curriculum as being unengaging, often due to its delivery format. As one student remarked, “It was online, and we had to write an essay at the end. It just wasn’t interesting.” Many students emphasized the need for interactive discussions in their PH education, with one stating, “I don't think it can be taught via pre-recorded lectures.”

Some noted that PH modules had fewer credits compared to other modules, leading them to delay coursework as a “strategic decision.” One student described how students “brushed it aside until the last minute.”

A common view was that medical education focused heavily on individual disease management rather than on disease prevention, or addressing broader health determinants. One student described how “medical school really focuses on medical problems you see in front of you and how to solve them. It doesn't really do a good job at addressing the person within (their) social, economic, and geopolitical context.”

#### 3.2.2 Subtheme 2.2: Lower prioritization by students compared with medical sciences

Students almost universally agreed that PH was lower on their list of priorities during their medical education. One prominent reason was the overwhelming volume of information they needed to absorb from the medical sciences. One student summarized the challenge of being a medical student as “just a survival of getting through.” Another participant noted that PH was deprioritized “because of the amount of stress that people had around the basic sciences and didn't have the time to put the same emphasis onto the PH module."

Many students believed that the lower prioritization of PH by students stemmed from the perception that it was not as relevant for medical students as the basic sciences. One participant stated, “there's that perception that PH is something that not everyone is going to be involved with, or it might not be as useful for everyone's practice, which ranks it lower.”

### 3.3 Theme 3: Limited exposure to PH in practice

#### 3.3.1 Subtheme 3.1: Limited opportunities for exposure to PH practice

Students almost universally described receiving limited exposure to PH practitioners during their medical studies, with many students never having met a PHM physician. Some students were unsure whether PHM was a distinct medical specialty. One student stated, “You get very little exposure [to PHM] beyond the epidemiology of a disease.”

Consequently, most students felt that they had little to no understanding of what the role of a PHM physician was. This was summarized by one student, who stated, “I couldn't visualize what a day-to-day life of a PH physician looked like.”

Students consistently highlighted their low exposure to PHM and the need to proactively actively seek out experiences. Two students had taken their own initiative to seek elective or observership opportunities with PHM physicians. Those who completed such opportunities reported that this practical exposure helped to shape their understanding of PHM as a distinct specialty and it placed their theoretical PH knowledge into a more practical medical context. One student reported, “if you don't go out and pursue PHM early on, then you just won't get the actual practical exposure to it in any real sense.”

Another student who completed an elective in PHM emphasized the value of practical exposure, reflecting how the elective had given them “a clearer insight into how practical a lot of PH can be.” While most students reported that they would not choose to undertake an elective in PHM if given this option, some believed that a one- or two-week placement would be beneficial during their medical studies.

#### 3.3.2 Subtheme 3.2: Reduced practical exposure impacts future career intentions

Many students cited their limited exposure to PH role models and to PH workplaces as factors that deterred them from considering PHM as a future career choice. One student reflected, “I think that could be because I'm not exposed so maybe I don’t understand [PHM] concepts or ideas as well as I could have.”

Several students believed that practical exposure to the specialty, or closer interaction with PHM physicians, could potentially change their attitudes and career decisions.

Even among the few students who were seriously considering a career in PHM, there was a self-rated poor understanding of career pathways. As one student with a strong interest in PH stated, “I am not even sure if there's a training scheme, per se, specifically looking at that, or if there's a role for that.”

## 4 Discussion

This study explored the perceptions of Irish medical students regarding PHM, their understanding of the specialty, and their expectations around incorporating PH principles in to their future clinical careers. Our findings highlighted key challenges in integrating public health practice in to medical education. Understanding medical students' attitudes toward PH education and practice is crucial for improving future recruitment in to the specialty.

The themes identified through interviews were broadly aligned with our scoping review of the international literature, particularly around misconceptions about PHM and limited opportunities for practical exposure to the specialty.

Our findings demonstrated mixed views among students on the relevance of PH education in medical curricula. Most participants acknowledged the relevance of PH principles in their training and education, but a minority remained skeptical.

Participants largely demonstrated a lack of understanding and knowledge of PH in our study and rated their own understanding of PH as low, contrasting with American students who felt more competent in their knowledge of PH principles [14]. This difference could stem from variations in curriculum delivery or due to broader educational and systemic differences. In U.S., the Association for Prevention Teaching and Research's Healthy People Curriculum Task Force (HPCTF) promotes PH education standards, while in Ireland, PH curriculum development is more decentralized [15].

Many students perceived PH as being peripheral to core clinical skills, often associating PHM with academic epidemiology and statistics rather than with any practical applications. Confusion about the role of PHM physicians was common, with students conflating their responsibilities with practitioners from other specialties such as general practice or infectious diseases.

Students perceived PH as being deprioritized in medical curricula, and some described delivery formats and assessments as unengaging. The perceived disconnect between PH coursework and its practical application hindered students' understanding of how PH knowledge translates into clinical practice. Students tended to prioritize other core scientific subjects over PH, particularly in the pre-clinical years, which may further obscure the role of PHM physicians.

Several participants expressed a clear desire for greater hands-on exposure to PH practice. This aligns with previous Canadian studies that report dissatisfaction with PH curricula due to limited availability of practical experiences and role models [16,17]. The importance of this practical exposure is further supported by a cross-sectional American study which found that the majority of medical students valued major PH initiatives and sought additional education in PH beyond their current curriculum, suggesting a demand among medical students for integrating PH principles more meaningfully into their clinical training [18].

Career interest in PHM varied, with minimal practical exposure contributing to students’ uncertainty about the specialty. Several students had never met a PHM physician and they harboured misconceptions or poor self-rated understanding of the role. Despite the overall low prioritization of PH in education at both curricular and personal levels, some students were eager to learn more. Mentorship and role models have a significant influence on medical student career trajectories, with cross-sectional studies highlighting the contribution of role models to shaping medical students career interests and specialization [[19], [20], [21]]. An additional longitudinal study across 24 medical schools in the USA found that personal exposure to a role model predicted choosing the role model’s specialty following graduating medical school [22]. Structured opportunities in PHM could foster medical student latent interest, as well as acquiring role models who may influence future career interest in PHM.

### 4.1 Implications

This study has some important implications for the role of PH in medical education going forward. It suggests the need for structured clinical placements and elective opportunities in PHM, as well as modifications to medical school curricula, to enhance medical students’ understanding of the specialty in Ireland. These may be facilitated by ensuring that PH practitioners, working within the health service, are more meaningfully involved in the delivery of the PH curriculum to medical students, ideally through formalised agreements or appointments within medical schools. Modifying medical school curricula to better engage students with PH could foster interest in PHM and better prepare future physicians for broader PH challenges in their future careers, regardless of specialty. Adopting structured PHM placements and greater practitioner involvement aligns with international objectives set by major health organizations in integrating public health into medical education in order to equip physicians with public health competencies to meet global health challenges [23]. By embedding these principles within medical curricula, Ireland – as well as other countries - can align its medical education system with global standards, ensuring its future physicians are prepared to promote health equity and disease prevention across all healthcare settings. The ASPHER organization has a role in aligning the PH curriculum in European institutions, encompassing undergraduate and postgraduate medical education [1]. A centralized task force in Ireland should be considered to coordinate the delivery of PH education across the medical universities, and in line with international standards.

Efforts in countries like Canada, the USA, the UK, and Ireland aim to standardize PH curricula and increase exposure to PH through elective experiences. [15]^,^ However, there has yet to be a structured integration of practical exposure to PHM in the medical curriculum [23]. In Portugal, PH seminars and clerkships are integrated directly into the medical curriculum, and this has shown demonstrable success in increasing student understanding of PH services [24]. Adapting such a model in Ireland may also help to address concerns about the lack of practical exposure to the specialty.

### 4.2 Strengths and limitations

This study provides valuable insights into medical students' perceptions of PH, being the first conducted in the Irish context. The qualitative approach herein allowed for in-depth exploration of students’ attitudes, while the diverse sample recruited from multiple universities strengthened the credibility and trustworthiness of the findings.

Limitations include the predominance of graduate-entry students, which may not fully represent the broader medical student population in Ireland. Graduate-entry students were disproportionately represented, likely because they were more responsive to recruitment emails and social media outreach efforts. They tend to be older in age, and may be more engaged with research participation, or may have more familiarity with core PH concepts from previous undergraduate experiences. This may limit transferability of our findings. Recruitment and response bias may have led to an overestimation of interest in PHM, although many students expressed negative attitudes to the specialty. The online setting may have also limited the capture of non-verbal cues during interviews, although the topics for discussion were not especially sensitive.

We were guided by the principles of information power, and we considered that our sample would be sufficient to meet our broad, exploratory aims. However, we employed cross-case thematic analysis which may have warranted a slightly larger sample size [13]. Future research on this topic should strive to ensure greater representation of undergraduate medical students where possible.

### 4.3 Conclusion

Medical students in Ireland perceive PH education as being of lower priority compared to other clinical modules. Participants perceived that PH was deprioritized by institutions and by students themselves. They have mixed views on its relevance to their future careers. Some students initially considered that PH seemed irrelevant to their medical education; yet, they simultaneously acknowledged that the core principles of PHM were important for all physicians to be aware of. Students have a limited understanding of PHM as a specialty, receive little exposure to PH workplaces, and report poor visibility of PH role models in the Irish context. These findings underscore the need to address pervasive misconceptions about PHM in practice, and provide more tangible opportunities for medical students to learn about the profession through clinical placements, elective opportunities, and curricular reform.

## Ethical statement

Ethical approval was obtained from the Social Research Ethics Committee and the Research and Postgraduate Affairs Committee at University College Cork. Written informed consent from all participants was collected at the start of each interview.

## Funding

The authors did not receive funding support from any organization for the submitted work.

## Declaration of competing interest

The authors declare no competing interests.
